# A Specific Role of Hippocampal NMDA Receptors and Arc Protein in Rapid Encoding of Novel Environmental Representations and a More General Long-Term Consolidation Function

**DOI:** 10.3389/fnbeh.2019.00008

**Published:** 2019-02-26

**Authors:** Cameron M. Bye, Robert J. McDonald

**Affiliations:** Canadian Centre for Behavioural Neuroscience, University of Lethbridge, Lethbridge, AB, Canada

**Keywords:** NMDA receptor, hippocampus, spatial learning, consolidation, Morris water maze

## Abstract

Activation of the NMDA receptor (NMDAR) has been proposed to be a key event responsible for the structural changes that occur in neurons during learning and memory formation. It has been extensively studied yet no consensus has been reached on its mnemonic role as both NMDAR dependent and independent forms of learning have been observed. We investigated the role that hippocampal NMDAR have in rapid spatial learning and memory across training environments. Hippocampal NMDAR was blocked via intra-hippocampal injection of the competitive antagonist CPP. Groups of rats were pre-trained on a spatial version of the Morris water task, and then mass reversal training under NMDAR blockade occurred in the same or different training environments as pre-training. We measured expression of Arc protein throughout the main hippocampal subfields, CA1, CA3, and dentate gyrus, after mass-training. We observed that NMDAR blockade allowed for rapid spatial learning, but not consolidation, when the SUBJECTS used previously acquired environmental information. Interestingly, NMDAR blockade impaired rapid spatial learning when rats were mass-trained in a novel context. Arc protein expression in the dentate gyrus followed this pattern of NMDAR dependent spatial behavior, with high levels of expression observed after being trained in the new environment, and low levels when trained in the same environment. CPP significantly reduced Arc expression in the dentate gyrus. These results implicate dentate NMDAR in the acquisition of novel environmental information.

## Introduction

The plasticity and the neurobiological mechanisms underlying memory-based behavior mediated by the hippocampus and related networks is complex and not completely understood. One neural property proposed to support the formation of memories is synaptic plasticity. Synaptic plasticity is the strengthening or weakening of synaptic connections between neurons via long-term potentiation (LTP) and long-term depression (LTD), respectively. LTP is an amplification of the excitatory post synaptic potential as a result of high frequency inputs (Bliss and Lømo, 1973; Levy and Steward, 1979). Typically, LTP is induced artificially through electrical stimulation but some of the biomarkers for LTP have been found in freely learning animals (Whitlock et al., 2006).

*N*-methyl-D-aspartate receptors (NMDAR) are a class of postsynaptic glutamate receptors located in many regions of the brain including the hippocampus (Dingledine, 1983). Although not necessary for normal synaptic transmission, the NMDAR plays an important role in mediating synaptic plasticity (Collingridge et al., 1983; Harris et al., 1984). By manipulating extracellular ion concentrations involved in NMDAR function like calcium or magnesium, LTP can be inhibited (Dunwiddie and Lynch, 1979; Herron et al., 1985). LTP can also be inhibited with NMDAR antagonists that block the receptor (Herron et al., 1986; Abraham and Kairiss, 1988). Genetic knockouts for the NMDAR in mice have also been associated with reduced synaptic plasticity and LTP in the hippocampus (Sakimura et al., 1995). A significant body of evidence over the past decades has shown a strong relationship between the expression of LTP and the NMDAR.

In a breakthrough study linking NMDAR function, LTP, and hippocampal based memory, Morris et al. (1986) bilaterally infused an NMDAR antagonist (APV) into the ventricles of rats that blocked LTP in the hippocampus. When trained on the Morris water task (MWT) that requires the rats to locate and learn the position of a hidden platform under a pool of opaque water, they failed to accurately find the hidden escape platform location. These results are similar to those of rats with hippocampal lesions (Sutherland et al., 1983). NMDAR inactivation has been associated with impairments in other learning tasks as well (Tonkiss et al., 1988; Ward et al., 1990). NMDAR antagonists have not been found to influence the retrieval of already formed memories (Shapiro and Caramanos, 1990). This early research has generated the popular theory that these receptors are critical for inducing synaptic plasticity, and therefore are critical for the encoding of experiences and memories (Collingridge and Bliss, 1987).

However, separate lines of research suggest that this story is not as straightforward as it might seem. NMDAR antagonists may have multiple effects on behavior because when administered intraperitoneally or intraventricularly (Morris et al., 1986; Morris, 1989; Ward et al., 1990) NMDARs are blocked throughout the entire brain (Monaghan and Cotman, 1985). Not all brain regions are primarily involved in memory functions and so blocking NMDARs in these areas may produce confounding behavioral effects. NMDAR antagonists have been shown to induce a wide variety of electrophysiological and behavioral impairments involving sensory (Salt, 1986), motor, (Cain et al., 1996), and anxiolytic responses (Stephens et al., 1986). For example, in the MWT motor impairments can severely limit the rats’ ability to learn (Cain et al., 1996). On top of all this, NMDAR independent LTP has also been found (Grover and Teyler, 1990).

Several behavioral methodologies have been proposed to avoid these potential confounds. Non-spatial visual discrimination tasks can be used to dissociate the effects that NMDAR antagonists might have on sensorimotor processes from their effects on learning. Typically, rats with NMDARs blocked are impaired on the MWT but successful at the visual discrimination task (Morris, 1989). More surprisingly, pre-training rats on the MWT has been shown to eliminate the learning deficits associated with NMDAR antagonists (Bannerman et al., 1995; Saucier and Cain, 1995). Briefly, if a rat is procedurally trained to learn the MWT, prior to drug administration and standard training, the rat is capable of learning new spatial information independent of NMDAR function at control levels. This pre-training is thought to diminish the potential impacts of the antagonist on sensory or motor functions.

Pre-training can take the form of standard training involving finding a hidden platform (McDonald et al., 2005), training in an entirely different context (Bannerman et al., 1995) or navigating the pool in a non-spatial way with either curtains drawn or a non-fixed platform position (Cain et al., 1996; Hoh et al., 1999). Studies utilizing pre-training have produced results very different from the earlier research and suggest that the proposed role of NMDAR in the acquisition of information may be incorrect.

Much of the spatial training that rats undergo while being administered NMDAR antagonist occurs over multiple days, however, if training occurs rapidly within a short time period then the rats may be able to acquire the spatial information (McDonald et al., 2005). Research using time delays suggests that the NMDARs role in memory may be in the consolidation of that information as opposed to its acquisition, a process that occurs later in time. The distinction between rapidly acquired information versus information that is acquired over days may explain the inability of rats to learn in several NMDAR antagonist studies. Indeed, the majority of research showing learning impairments following blockade of NMDAR in spatial memory are done over several days of drug administration (Morris, 1989; Robinson et al., 1989; Inglis et al., 2013).

Critical support for the consolidation idea came from Kentros et al. (1998). They examined the effects that a NMDAR antagonist would have on the formation and stability of place fields in the hippocampus. They found that NMDAR blockade did not prevent the formation of new place field representations in the hippocampus when the rat was located in a new environment and the representation lasted for approximately 1.5 h. The antagonist did, however, prevent the long-term stability of the representation. This study provided important electrophysiological support for the idea that the acquisition of new spatial information is possibly independent of NMDAR function.

Further support came from McDonald et al. (2005) who pre-trained rats to find a hidden location in the MWT, then later rapidly trained to a new location while given intraperitoneal or intra-hippocampal injections of a NMDAR antagonist. The rats successfully learned the new location during the training period showing acquisition without NMDAR function. When tested 24 h later, the rats did not remember what they had learned suggesting that their ability to learn was intact while their ability to consolidate the information into long-term memory was impaired.

**FIGURE 1:**
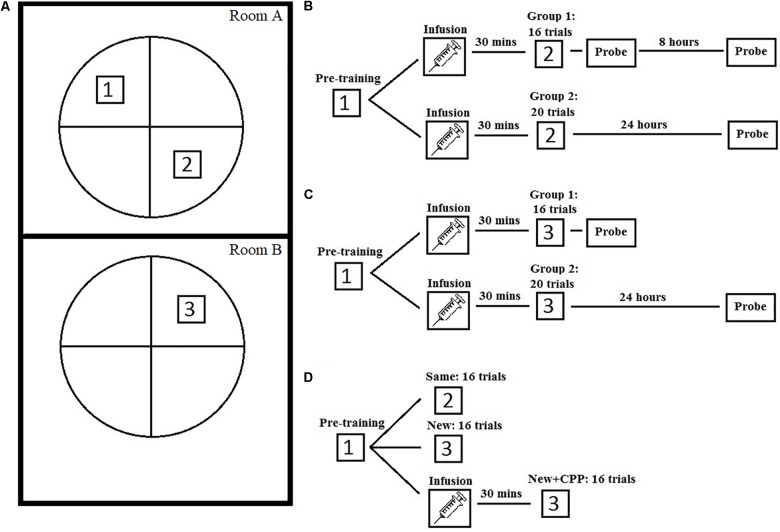
Study design of the three experiments. **(A)** A graphic representation of the water task setup and platform in both experimental rooms. **(B)** A schematic of the study design for experiment 1. **(C)** A schematic of the study design for experiment 2. **(D)** A schematic of the study design for experiment 3.

The goal of the present work is to further assess the role of NMDARs in hippocampal-based learning and memory functions. An outline of these experiments can be found in Figure 1. Experiment 1 was an extension of the work done by McDonald et al. (2005) in which an NMDAR antagonist was only administered to the dorsal hippocampus, leaving the ventral portion most likely unaffected although the exact drug diffusion is unknown. It is possible that ventral hippocampal NMDA-based plasticity could support place learning which could account for the lack of effect in this study. From an experimental design perspective, intra-cranial injections of NMDA directly into the entire hippocampus is also important because this procedure leaves NMDARs in other brain regions unaffected allowing the isolation of the mnemonic effects of this manipulation from any other potential behavioral effects.

For Experiment 1, rats were given 4 days of spatial pre-training, followed by NMDAR antagonist drug infusion (2-carboxypiperazin-4-yl)-propyl-1-phosphonic acid (CPP) and mass spatial training to a new platform location. This was followed by a series of probe tests, one immediately after training to test the hypothesis that hippocampal NMDARs are necessary for the acquisition of novel spatial information, and two others done 8 h and 24 h later to examine the effect of NMDAR blockade on short-term memory consolidation. Based on our previous results we hypothesized that rats should be able to acquire new information without the use of NMDARs, but that this information would not be consolidated and would disappear after a short period of time.

## Materials and Methods

### Subjects, Acclimation, and Handling

Subjects were male Long Evans rats aged 90 days upon arrival to the facility (*n* = 16). The weight range at the start of the experiment was between 300 and 350 g. They were housed in pairs and were kept on a 12 h light/12 h dark cycle with lights turning on at 7:30 and turning off at 19:30. The rats had *ad libitum* access to both water and food. Rats were allowed 7 days of acclimatization in their home cages to reduce stress induced from travel. After this period, all rats from each experiment were handled for 5 min a day for 5 days to familiarize them with the experimenters and being manipulated. All procedures were in accordance with the regulations set out by the Canadian Council of Animal Care and approved by the University of Lethbridge Animal Care Committee.

### Training Room/Apparatus

A large white circular fibreglass pool 46 cm in height and 127 cm in diameter was used. The pool was placed roughly in the center of the room. The pool was filled with water low enough that the rats could not escape by climbing onto the walls but high enough that the rats could see the extra maze cues on the laboratory walls. Water was made non-transparent with non-toxic white paint. The pool was emptied, cleaned, and refilled with fresh water daily. The escape platform was located approximately 2 cm below the surface of the water. It was a white plastic circle 13 cm in diameter and made up approximately 1% of the total surface area of the pool. It had several small holes drilled into the surface for grip. Posters of simple colored geometric shapes were placed on the walls of the laboratory room to serve as visual cues along with the computer, experimenter, a large black shelf, and a door.

### Surgery

Permanent guide cannulae were implanted bilaterally into the hippocampus of all rats. Rats received subcutaneous injections of buprenorphine (Temgesic^®^, Schering-Plough) at 0.03 mg/kg prior to surgery to offer post-surgical analgesia. Rats were anesthetized using 4% isoflurane gas (Benson Medical Industries, Inc.) in oxygen with a flow of 1.5 l/min. Surgical anesthetic plane was maintained using 1–2% isoflurane throughout surgery. The rats were positioned in a stereotaxic apparatus (Kopf Instruments). An incision was made in the scalp, the skin retracted, and seven 0.7 mm holes were drilled into the skull. Three pilot holes were drilled for anchor screws (Small Parts, United States), and four holes for guide cannulae. Two 23-gauge stainless steel guide cannulae were lowered bilaterally into the dorsal (A/P -3.5, M/L: ±2.0, D/V: -3.2) and ventral (A/P: -5.8, M/L: ±5.2, D/V: -6.0) hippocampus and were held in position using dental acrylic. The guide cannulae were plugged using 30-gauge wire obturators, which stayed inside until infusion. Following surgery, rats were injected with Metacam^®^, 5 mg/ml, 0.5 mg/kg (Boehringer Ingelheim) and monitored for 24 h, then returned to their home cages.

### Data Collection and Statistical Analysis

Data were collected with a movement tracking software (Noldus Ethovision 3.1) and a ceiling mounted camera. Statistical analysis was performed using IBM SPSS 34 Statistic Version 22. Acquisition and probe test data were analyzed with two-way repeated measures ANOVA. When a significant interaction occurred, planned *post hoc* pair wise comparisons were done between the pre- and mass trained quadrants on the trial 1 and immediate probes as we expected differences to occur. Planned *post hoc* pairwise comparisons were done for the consolidation probes comparing % time spent in the pre- vs. mass trained quadrants.

### Training/Testing

The training procedure consisted of a three phase version of the MWT. All training and testing occurred in the same room and occurred between 07:00 and 12:00, except for phase 3. Two groups of rats were run in this experiment, differing only in the amount of training and time between phases.

#### Phase 1

Rats were brought into the testing room in individual cages on a wheeled cart and placed into the NE corner of the room. Animals were run in squads of 4, one right after the other. For this phase, all rats were trained to find a hidden platform located in the SW quadrant of the pool. Each rat was given eight trials a day for 4 days, for a total of 32 trials. The starting position of each trial was randomly assigned to arbitrarily equidistant points. The sequence of start points varied each day. The rat was placed in the pool at one of the start positions facing the pool wall and allowed to swim until they reached the hidden platform or until 60 s had elapsed. If after 60 s the rat had not found the platform it would be led there by hand. After every trial the rat would be left on the platform for 10 s, removed and placed back in its transport cage. No drugs were administered during this phase of training. Each training session took approximately 30–40 min with an average inter-trial interval of 5 min.

#### Phase 2

Twenty-four hours after completing phase 1 rats began phase 2 of training. The platform was moved to the NE quadrant, opposite to that of phase 1. For group 1, training consisted of 16 trials within a 2-h period, all on day 5. For group 2, training was 20 trials. Similar to phase 1, rats were placed in the pool at one of the cardinal positions in random order, were allowed 60 s to find the platform, and remained on it for 10 s. The NE starting point was eliminated during phase 2 because it was closest to the platform. Prior to training, rats were brought into a novel room and assigned to either the treatment group or a control group. The assignment to treatment groups was designed in such a way that there was no difference in the pre-training acquisition between pre-treatment groups. The treatment group (group 1: *n* = 6, group 2: *n* = 7) received bilateral hippocampal infusion CPP in artificial cerebral spinal fluid (0.32 ng/μl). The control group (group1: *n* = 7, group 2: *n* = 7) received just artificial cerebral spinal fluid. Obturators were removed and infusions were done at a rate of 0.25 μl/min for 4 min, for a total of 1 μl per infusion site. This dose is the same used in the McDonald et al. (2005) study and is a dose that has been shown to impair spatial performance (Riekkinen and Riekkinen, 1997).

The infusion cannulae were left inside the permanent guide cannula for an extra minute to allow for diffusion of the drug. After this 5-min procedure, new obturators were placed into the permanent guide cannula and rats were returned to their home cage. Training began 30 min after infusion.

For group 1, the platform was removed after the 16 massed training trials and a 30-s probe test was given. The interval between the last trial and the probe test was 5 min. Group 2 did not receive a probe test during this time. All training occurred within a 2-h period, a time frame that CPP has been shown to block prime-burst potentiation in the hippocampus (Kentros et al., 1998).

#### Phase 3

Eight hours after completing the phase 2 probe test, group 1 received a second probe test. This was done to determine if what was learned during mass training would be remembered. The rat was placed in the pool in the exact same way, in the same start location, as the prior probe test. After 30 s the rat was removed from the pool. Group 2 received a probe test 24 h after completing phase 2. This difference in the two groups was used to examine potential differences in periods of consolidation.

### Perfusion

The day after phase 3 the rats were euthanized with an intraperitoneal injection of sodium pentobarbital (300 mg/kg) and then transcardially perfused with 4% paraformaldehyde solution and 5% phosphate buffered ACSF. The tissue was left in the 4% paraformaldehyde solution for 24 h for cryoprotection, then placed into a 30% sucrose + 0.2% sodium azide solution for 5 days. Brain tissue was sliced on a freezing microtome and sections of the hippocampus were stained with a cresyl violet stain. Proper cannulation placement was analyzed and all subjects with cannulation points outside of the hippocampus were excluded from analysis.

## Results

### Experiment 1

An outline of Experiment 1 can be found in Figure 1.

#### Results Group 1

**FIGURE 2:**
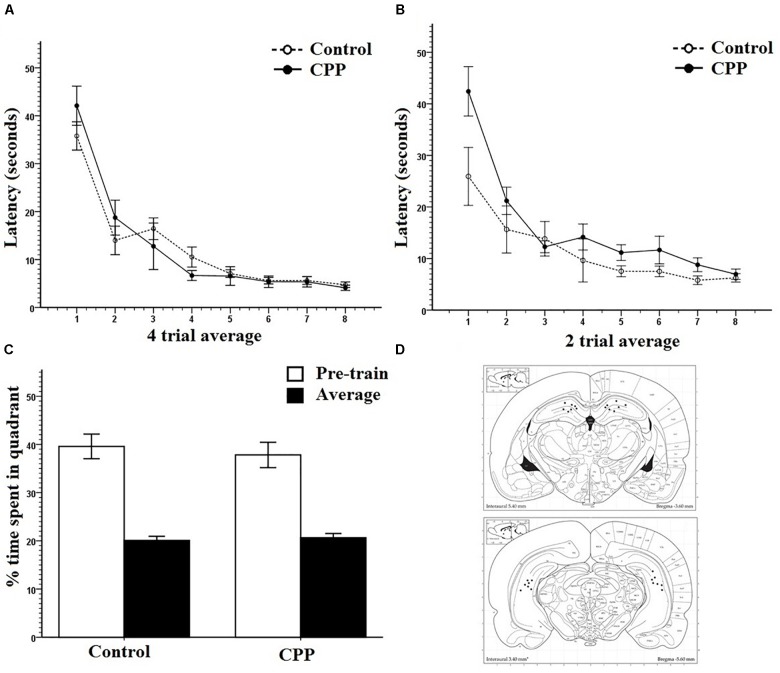
**(A)** Pretraining for group 1 in Experiment 1. Four trial averages of the latency in seconds to find the platform over training 4 days/eight trials per day. Both controls and pre-CPP rats successfully learn over training. **(B)** Mass training for group 1 in Experiment 1. Two trial averages of the latency in seconds to find the new platform over training 2 h/16 trials. Both controls and CPP rats learn over training. **(C)** A probe test of trial 1 mass training in group 1 of Experiment 1. The percentage of time spent of the trial length in the pre-training quadrant versus an average of the other three. Both controls and CPP rats display a preference for the pre-trained quadrant. **(D)** Dorsal and ventral hippocampal cannulation locations for the CPP rats of group 1 in Experiment 1. Error bars 1 ± SE.

The primary measure of learning and memory used during acquisition was latency to find the platform. Path length was also recorded with identical results across all experiments and so were not included for sake of space. However, they can be found in the Supplementary Material. Pre-training was analyzed in four trial average blocks. Figure 2A clearly shows that over the 4-day pre-training period, all rats from both groups learned the escape platform position in the pool. Two-way repeated measures ANOVA showed that there was a significant effect of Trial (*F*_7,77_ = 63.307, *P* < 0.001) on latency but no effect of Group (*F*_1,11_ = 0.016, *P* > 0.05) and no interaction (*F*_7,77_ = 1.571, *P* > 0.05). Pre-training data were reliably identical across groups and experiments and so will only be presented as a graph once (Figure 2A).

Figure 2B shows that with or without hippocampal NMDAR function, rats successfully learned a new platform position over a 2-h training period. Mass-training was analyzed in two trial average blocks. Despite the initial difference in escape latency, a two-way repeated measures ANOVA revealed a significant effect of Trial on latency (*F*_7,77_ = 22.829, *P* < 0.001) but not of Group (*F*_1,11_ = 4.008, *P* > 0.05) and no interaction (*F*_7,77_ = 1.984, *P* > 0.05).

To determine if the rats had successfully acquired a spatial memory during pre-training, as well as test the effects of CPP on the expression of already formed memories, the first trial of mass training was analyzed as a probe trial (Figure 2C). Comparisons were made between the percentage of time spent in the target quadrant where rats were trained during pre-training and an average of the percentage of time spent in the other three quadrants. Because the platform was present during this trial, not all animals had equal latencies on trial one of mass-training and so not all animals spent an equal amount of time searching within the pool. The percent of time spent in each quadrant given the total time each animal spent in the pool was used. Controls spent an average 39.6% search time in the target quadrant and 20.1% in all other quadrants. CPP infused rats spent an average 37.8% search time in the target quadrant and an average of 20.7% in all other quadrants. Two-way repeated ANOVA revealed that there was a significant effect of quadrant (*F*_1,11_ = 55.660, *P* < 0.001) but not of Group (*F*_1,11_= 0.247, *P* > 0.05) and no interaction (*F*_1,11_ = 0.227, *P* > 0.05). These data show that rats had developed a spatial memory during pre-training and that CPP did not interfere with the expression of this memory. Cannulation placements for this experiment can be seen in Figure 2D.

**FIGURE 3:**
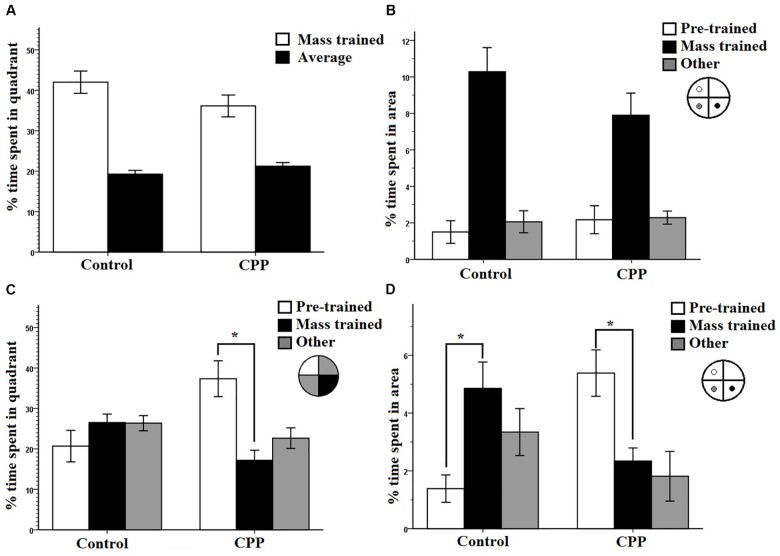
**(A)** A 30-s probe test immediately after mass training for group 1 in Experiment 1. The percentage of time spent in the mass trained quadrant versus an average of the other three was compared. Both controls and CPP rats displayed a preference for the mass trained quadrant. **(B)** A 30-s probe test immediately after mass training for group 1 in Experiment 1. The percentage of time spent in the region immediately surrounding the platform locations during pre-training, mass training, and a non-trained area were compared. Both controls and CPP rats displayed a preference for the area surrounding the mass trained platform. **(C)** A 30-s probe test 8 h after mass training for group 1 in Experiment 1. The percentage of time spent in the pre-trained quadrant, mass trained quadrant and the average of the other two was compared. Controls displayed no preference whereas CPP rats displayed a preference for the pre-trained quadrant. **(D)** A 30-s probe test 8 h after mass training for group 1 in Experiment 1. The percentage of time spent in the region immediately surrounding the platform locations during pre-trained, mass trained and a non-trained area were compared. Controls displayed a preference for the area immediately surrounding the mass trained platform whereas CPP rats displayed a preference for the area surrounding the pre-trained platform. Error bars 1 ± SE, ^∗^*p* < 0.05.

After 16 trials of mass-training to the new quadrant location, the platform was removed, and rats were put through a 30-s probe test to determine if a new spatial preference had been learned (Figure 3A). Comparisons were made between the percentage of time spent in the new target quadrant where rats were trained during mass training and an average of the percentage of time spent in the other three quadrants. ACSF infused controls spent an average of 42% in the new target quadrant and 19.3% in the all other quadrants. CPP infused rats spent an average of 36.1% in the new target quadrant and 21.2% in all others. Two-way repeated measures ANOVA revealed a significant effect of quadrant (*F*_1,11_ = 52.568, *P* < 0.001) but not of Group (*F*_1,11_= 2.289, *P* > 0.05) with no interaction (*F*_1,11_ = 2.280, *P* > 0.05). These results indicate that both groups had learned a new spatial preference following mass-training.

Probe data were also analyzed comparing percentage of time spent in a small region surrounding the platform covering 2% of the total surface area of the pool (annulus), to contrast with the 25% surface area of the quadrant. This type of analysis provides information about the spatial specificity of what was learned during mass training, as the region of interest is limited to the area immediately surrounding the platform location. Controls spent an average of 10.2% in the new annulus and an average of 1.5% in the pre-trained location. CPP infused rats spent an average of 7.9% in the new annulus quadrant and an average of 2.2% in all others (Figure 3B). Two-way repeated measures ANOVA revealed a significant effect of quadrant (*F*_2,22_ = 51.402, *P* < 0.001) but not of Group (*F*_1,12_= 0.355, *P* > 0.05) with no interaction (*F*_2,22_ = 2.090, *P* > 0.05). This shows that the spatial preference rats learned over training was specific to the platform location.

Previous work has shown that hippocampal NMDARs may have a critical role in the consolidation of newly acquired memories (McDonald et al., 2005; Roesler et al., 2005). To assess this claim, for group 1, a probe test was administered 8 h after the end of mass training to determine if what was learned during mass-training would remain or be forgotten due to a lack of consolidation.

The percentage of time spent in the two trained target quadrants, pre-training and mass training, as well as the average percentage of time spent in the other two non-trained quadrants, were compared within and between groups (Figure 3C). Two-way repeated measures ANOVA, showed no effect of quadrant (*F*_2,22_ = 2.043, *P* > 0.05), no effect of Group (*F*_1,11_ = 11.373, *P* > 0.05), but a significant Group × Quadrant interaction (*F*_2,22_ = 7.256, *P* < 0.05). *Post hoc* pairwise comparisons using LSD revealed a significant difference within the CPP group with these rats spending more time in the pre-trained quadrant (Avg = 37.35%) then in the mass trained quadrant (Avg = 17.18%) (*P* = 0.03) No difference was found within the control group between any of the quadrants.

These results indicate that the effect of mass training did not last 8 h in the rats given CPP, and that their spatial preference reverted back to the pre-trained quadrant. The control probe data seems harder to interpret. On the surface this quadrant preference result would suggest that the control rats had forgotten the mass-trained target platform position. However, we do not think this is the case. We were concerned that the 30-s probe trial performed immediately following mass training might have resulted in some extinction and provided the subjects with the knowledge that the platform might be located nowhere (probe trial) or elsewhere (mass training). The combination of this new knowledge and some extinction could have resulted in a somewhat contaminated competition test. We were intrigued by the idea that the control rats might have swam to the target annulus but did not perseverate there because of this new knowledge and some extinction. Importantly, this probe trial was not part of any of the experiments reported in McDonald et al. (2005) in which the control rats always showed a quadrant preference for the mass-trained location in different groups of rats over many experiments. One possibility was that the rats did search the mass-trained location but did not perseverate there for long.

To test this idea, we analyzed the 8-h probe trial in another way. This probe data was analyzed comparing percentage of time spent in the annulus of the pre-trained and mass-trained platform locations. Probe data for the 8-h consolidation probe was analyzed comparing percentage of time spent in the annulus of the pre-trained and mass trained platform locations. Controls spent an average of 4.8% in the mass trained annulus and an average of 1.3% in the pre-trained annulus. However, CPP infused rats spent an average of 2.3% in the pre-trained annulus and an average of 5.4% in the mass trained annulus (Figure 3D). Two-way repeated measures ANOVA revealed no significant effect of quadrant (*F*_2,24_= 1.029, *P* < 0.001) and not of Group (*F*_1,12_= 0.001, *P* > 0.05). However, there was a significant interaction (*F*_2,22_ = 10.936, *P* < 0.001). *Post hoc* pairwise comparisons using LSD revealed a significant difference within the CPP group, rats spending more time in the pre-trained location then in the mass trained location (*P* = 0.012). Control rats spent significantly more time in the mass trained location than in the pre-trained location (*P* = 0.006).

These results indicate that rats given CPP reversed their spatial preference compared to controls 8 h after mass-training suggesting that the CPP rats did not consolidate the representation guiding them to the mass training location. Although it appeared that the control rats did not remember the mass-training escape location based on the quadrant preference data, they clearly expressed a memory for the correct location and this memory was highly specific to the correct annulus providing direct evidence that they retained the mass-training representation for at least 8 h.

#### Results Group 2

Figure 1 shows the experimental design for Group 2. As in Group 1, latency and various measures of spatial specificity of behavior during probe trials were analyzed Group 2. For the initial acquisition data a two-way repeated measures ANOVA showed that there was a significant effect of Trial (*F*_7,84_= 26.965, *P* < 0.001) on latency but no effect of Group (*F*_1,12_ = 0.925, *P* > 0.05) and no interaction (*F*_7,84_ = 0.537, *P* > 0.05). Over the 4-day pre-training period, all rats from both groups learned the platform position in the pool.

**FIGURE 4:**
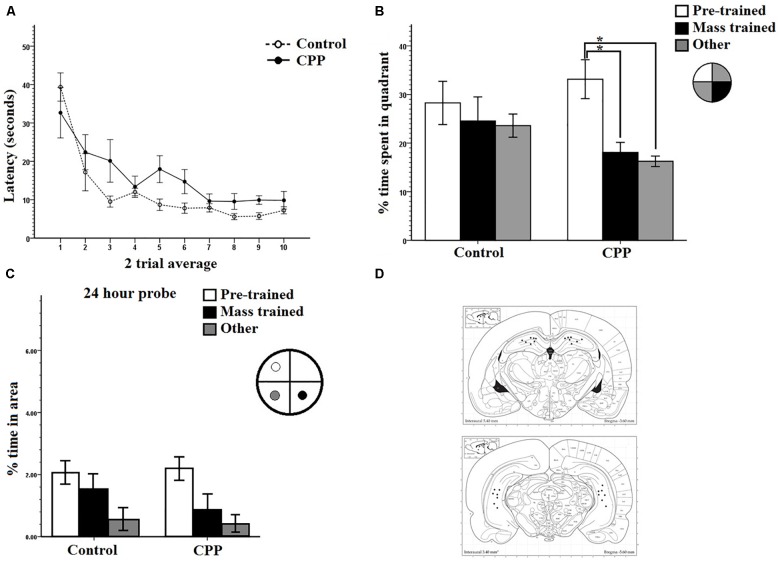
**(A)** Mass training for group 2 in Experiment 1. Two trial averages of the latency in seconds to find the new platform over training 2 h/20 trials. Both controls and CPP rats learn over training. **(B)** A 30-s probe test 24 h after mass training for group 1 in Experiment 1. The percentage of time spent in the pre-trained quadrant, mass trained quadrant and the average of the other two was compared. Controls displayed no preference whereas CPP rats displayed a preference for the pre-trained quadrant. **(C)** A 30-s probe test 24 h after mass training for group 2 in Experiment 1. The percentage of time spent in the region immediately surrounding the platform locations during pre-trained, mass trained and a non-trained area were compared. **(D)** Dorsal and ventral hippocampal cannulation locations for the CPP rats of group 2 in Experiment 1. Error bars 1 ± SE. ^∗^*p* < 0.05.

The data from the massed training day for Group 2 is shown in Figure 4A. As can be seen, rats with or without hippocampal NMDAR function could successfully learn a new platform position in the same training context over a 2-h period. Consistent with these observations, a two-way repeated measures ANOVA revealed a significant effect of Trial on latency (*F*_9,108_ = 17.898, *P* < 0.001) but not of Group (*F*_1,12_ = 4.106, *P* > 0.05) and no interaction (*F*_9,108_ = 1.412, *P* > 0.05). Cannulation placements for this experiment can be seen in Figure 4D.

For Group 2, the probe test for memory retention was done 24 h after completion of mass-training. The percentage of time spent in the two trained target quadrants, pre-training and mass-training, as well as the average percentage of time spent in the other two non-trained quadrants, were compared within and between groups (Figure 4B). Two-way repeated measures ANOVA, showed a significant effect of quadrant (*F*_2,24_ = 4.408, *P* < 0.05), a significant effect of Group (*F*_1,12_= 7.681, *P* < 0.05), and no Group × Quadrant interaction (*F*_2,24_ = 1.366, *P* > 0.05). *Post hoc* pairwise comparisons using LSD revealed a significant difference within the CPP group, rats in this group spent more time in the pre-trained quadrant (Avg = 33.1%) then in the mass-trained quadrant (Avg = 18.1%) (*P* = 0.05) as well as the average of the other two quadrants (16.3%) (*P* = 0.009). No differences were found within any of the control group percentages or between groups.

Just like for group 1, the control rats from group 2 did not appear to remember the rapidly acquired new location when tested later. For group 2, the subjects were tested 24 h later. Interestingly, when we did an alternative analysis of the 8-h probe data for group 1 in which we assessed the spatial specificity of the probe trial we found evidence that the controls did remember the precise location of newly acquired escape platform (spending more time in the new platform annulus versus the old platform annulus) whereas the CPP rats spent more time in the old platform annulus.

We did this same analysis for the 24-h probe groups and the results suggest that both the control and CPP rats did not remember the newly acquired location 24-h later (Figure 4C) but the CPP rats did seem to prefer the old annulus location. An ANOVA with repeated measures on location indicated a significant effect of swim location (*F*_2,24_ = 7.6401, *P* < 0.0027), but no Group (*F*_1,12_ = 0.7531, *P* = 0.4) or interaction effect (*F*_2,24_= 0.4261, *P* = 0.6). A *post hoc* pairwise comparisons using LSD indicated significant differences between the old and new platform location (*p* < 0.03), and the old and other location (*p* < 0.001).

**FIGURE 5:**
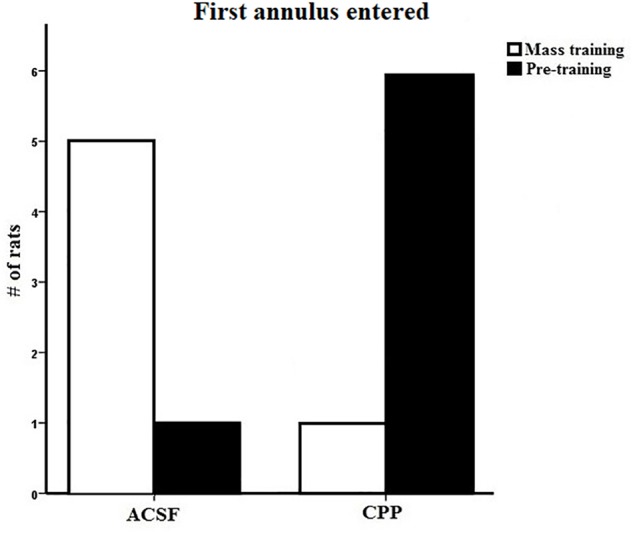
First annulus entered during the 24-h probe test for group 2 in Experiment 1. Five out of the six control rats first swam to the mass trained annulus where as six out of the seven CPP rats first swam to the pre-trained annulus.

We completed one final analysis of this data set looking at what annulus area each subject entered first during the probe trial. In the test of first annulus area entered for the 24-h probe, most of the controls swam to the mass trained annulus over the pre-trained annulus location (Figure 5) and the CPP rats first swam to the pre-trained escape annulus. We employed a chi-square analysis of this data set with the knowledge that this statistic requires high sample sizes to have enough power. Despite this caveat, the control preference was marginally significant (5/6, *X*^2^ = 5.6, *df* = 2, *P* = 0.060), whereas analysis of the CPP rats that swam to the pre-trained over the mass trained annulus was significant (6/7, *X*^2^ = 7.4, *df* = 2, *P* = 0.025).

The 24-h probe results are similar to those found in group 1 and indicate that rats with hippocampal NMDARs blocked can rapidly learn a new spatial position during mass training, they do not maintain this preference over a 24-h period. Control rats showed evidence that they did remember the new spatial position 24 h later by entering the new platform position first providing further evidence that hippocampal NMDARs are involved the consolidation of spatial memory.

#### Discussion

In this experiment, rats with bilateral hippocampal cannulations were pre-trained on the spatial version of the MWT, given either intra-hippocampal CPP or ACSF and then mass-trained to a new platform location.

The general pattern of results showed that rats with hippocampal NMDAR blockade were able to rapidly acquire information to help them navigate to a new spatial location in a pool in which pre-training to a different location had occurred. Probe trial data suggested that although this representation was acquired the CPP rats, it was not consolidated properly.

These results provide three insights into the role of NMDAR in learning and memory. Firstly, NMDAR are not necessary for the expression of previously acquired memories, and the blockade of NMDAR does not extinguish or alter previously acquired memories (Shapiro and Caramanos, 1990; Kim et al., 1991; Matus-Amat et al., 2007). Second, NMDAR are not necessary for the rapid acquisition of novel information. This effect has been explored in different ways in the past (Bannerman et al., 1995; Otnæss et al., 1999; Inglis et al., 2013) and compliments the previous work done in our lab (Holahan et al., 2005; McDonald et al., 2005). Third, NMDARs have a role in the consolidation of recently acquired memory (Kesner and Dakis, 1995; Santini et al., 2001).

### Experiment 2

It has been argued that in studies where the same training context is used for both pre-training and standard training (Hoh et al., 1999; McDonald et al., 2005), most of the learning that is crucial for completing the task occurs during the pre-training phase and that later training in the task can be completed without engaging plasticity mechanisms in the hippocampus. This means that even though the platform is in a different position during regular training, the rat can locate and “learn” the new position without needing hippocampal plasticity mediated by NMDARs and may either rely on previously acquired hippocampal memories or cortical plasticity (Inglis et al., 2013). However, even when two completely different contexts are used (Bannerman et al., 1995), replications have failed to reproduce identical results (Inglis et al., 2013). To determine whether NMDARs only encode novel spatial information, and not further learning dependent on previously acquired spatial information, a second version of Experiment 1 was completed but using two different contexts for pre-training and mass training.

#### Methods

In the multiple room version of the rapid acquisition MWT procedure, animals were pre-trained in room A: a separate room at the other end of the facility, with distinct extra maze cues and relationships between these cues. These included the door, posters, computer, and experimenter. This training consisted of 4 days of training, eight trials a day, to the same platform location each day, similar to Experiment 1. For the second phase, animals were mass-trained to a new platform location in room B over 2 h. Room B was the same room used in Experiment 1. The animals were brought to the room in a different style of transport cage between pre-training and mass-training phase. The drug administration process was identical to Experiment 1, either CPP or ASCF was infused bilaterally into the hippocampus 30 min prior to mass training in room B. A depiction of the two rooms and experimental design can be seen in Figure 1.

Two groups of rats were trained on this task. For group 1 (*n* = 13), mass-training consisted of 16 trials in room B. In group 2 (*n* = 11), mass training was 20 trials. Trial one of mass training was analyzed in a similar way as Experiment 1 to assess if spatial pre-training in room A had any effects on novel spatial navigation. Previous work from our lab has shown that some aspects of spatial navigation can transfer between training environments (Clark et al., 2015). Group 1 received a 30-s probe test immediately after mass training to assess whether mass training induced a spatial preference. For group 2, a 30-s probe test was run 24 h after mass-training to determine if what was learned during mass-training was consolidated into long-term memory.

Surgical procedure, data analysis, and histology was identical to that of Experiment 1.

#### Results Group 1

**FIGURE 6:**
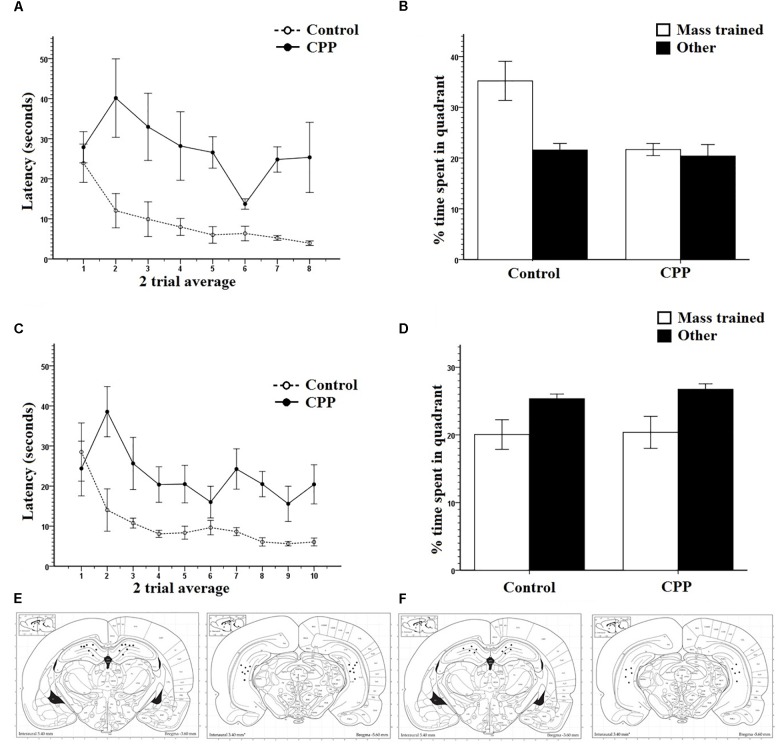
**(A)** Mass training for group 1 in Experiment 2 in room B after being pre-trained in room A. Two trial averages of the latency in seconds to find the new platform over training 2 h/16 trials. Control rats learned over training whereas as CPP rats did not. **(B)** A 30-s probe test immediately after mass training for group 1 in Experiment 2. The percentage of time spent in the mass trained quadrant versus an average of the other three was compared. Control rats displayed a preference for the mass trained quadrant whereas the CPP rats did not. **(C)** Mass training for group 2 in Experiment 2 in room B after being pre-trained in room A. Two trial averages of the latency in seconds to find the new platform over training 2 h/20 trials. Control rats learned over training whereas as CPP rats did not. **(D)** A 30-s probe test 24 h after mass training for group 1 in Experiment 2. The percentage of time spent in the mass trained quadrant versus an average of the other three was compared. No group of rats displayed a preference for mass trained quadrant. **(E)** Dorsal and ventral hippocampal cannulation locations for the CPP rats of group 1 in Experiment 2. **(F)** Dorsal and ventral hippocampal cannulation locations for the CPP rats of group 2 in Experiment 2. Error bars 1 ± SE.

Similar to the results of Experiment 1, both groups of rats successfully learned to navigate to the hidden platform location over the course of pre-training. A two-way repeated measures ANOVA showed that there was a significant effect of Trial (*F*_7,63_= 39.969, *P* < 0.001) on latency but no effect of Group (*F*_1,9_ = 0.704, *P* > 0.05) and no interaction (*F*_7,63_ = 0.704, *P* > 0.05). Figure 6A shows the mass-training data and clearly indicates that the control group learned the new location of the escape platform while the CPP group did not learn the new location over the course of training. A two-way repeated measures ANOVA revealed a significant effect of Trial on latency (*F*_7,63_ = 3.599, *P* < 0.05) and of Group (*F*_1,9_ = 19.872, *P* < 0.05) but no interaction (*F*_7,63_ = 1.856, *P* > 0.05).

The probe test run immediately after mass training showed that control rats spent more time in the quadrant that the new platform position was previously located while the CPP rats did not show this quadrant preference (Figure 6B). Control rats spent more time in the target quadrant (35.2%) than in the average of the other three (21.6%) (Figure 6B). This was not the case for the CPP rats who spent relatively equal time in each (21.6% and 20.5%). Consistent with these observations two-way repeated measures ANOVA revealed a significant effect of quadrant (*F*_1,9_ = 5.811, *P* < 0.05) and of Group (*F*_1,9_ = 15.975, *P* < 0.05) but no interaction (*F*_1,9_ = 4.179, *P* > 0.05). This pattern of results show that NMDAR blockade impairs rats’ ability to learn a spatial location when the available environmental information is novel.

#### Results Group 2

Both groups of rats successfully learned to navigate to the escape platform position during pre-training. For this data set, a two-way repeated measures ANOVA showed that there was a significant effect of Trial (*F*_7,77_ = 47.696, *P* < 0.001) on latency but no effect of Group (*F*_1,11_ = 0.423, *P* > 0.05) and no interaction (*F*_7,77_ = 0.587, *P* > 0.05). Figure 6C shows the mass training data and clearly shows that control rats learned the new escape platform position over the course of mass training whereas rats given intra-hippocampal CPP injections were severely impaired. Statistical analysis of this data set using a two-way repeated measures ANOVA revealed a significant effect of Trial on latency (*F*_9,99_ = 4.192, *P* < 0.001) and of Group (*F*_1,11_ = 11.5, *P* < 0.05) but no interaction (*F*_9.99_ = 1.842, *P* > 0.05).

For the 24-h probe, the percentage time swam in the quadrant where the platform was located during mass-training was compared to an average of the other three quadrants (Figure 6D). As can be seen (Figure 6D) both groups did not spend more time in the mass-trained quadrant compared to the other quadrants. A two-way repeated measures ANOVA revealed an effect of quadrant (*F*_1,11_ = 8.565, *P* < 0.05) but not of Group (*F*_1,11_ = 0.414, *P* > 0.05) and no interaction (*F*_1,11_ = 0.074, *P* > 0.05). These results suggest that massed training to a new location in a new room was not retained 24 h after training in either groups. The implication of this pattern of results will be discussed below.

Cannulation placements can be seen in Figure 6E,F.

#### Discussion

When rats were given intra-hippocampal CPP injections they were incapable of acquiring novel spatial information in room B, despite spatial pre-training in room A. Probe tests revealed that controls acquired a spatial preference for the pool quadrant where the platform was located, CPP rats did not. This contrasts with Experiment 1 in which rats were capable of temporarily acquiring new spatial information under hippocampal NMDAR blockade in the same room as pre-training. The only factor that differed from these two experiments was the spatial information available to the rats during mass training.

The results of Experiment 2 indicate that hippocampal NMDAR may be necessary for the acquisition of spatial information during MWT training (the arrangement and type of extra-maze cues, room and maze geometry). Presumably, this environmental information would be learned during pre-training in room A. Once this has been learned, however, hippocampal NMDARs would not be necessary for using this same information in learning new things, such as learning to navigate to a new platform location. When brought to a novel room, however, rats without hippocampal NMDAR function could not perform at the level of controls, indicating their importance in novel spatial learning.

When brought to training room B, both CPP and ASCF controls displayed a spatial preference for one of the pool quadrants during trial 1 of mass-training, despite having never been in room B before. This means that some information learned during pre-training in room A carried over to room B. It has been shown that some components of spatial navigation, namely heading direction, can be transferred between spatially distinct environments (Dudchenko and Zinyuk, 2005; Clark et al., 2015). Head direction cell orientation is often controlled by environmental boundaries and maze shape (Hamilton et al., 2007; Clark et al., 2012). Indeed, in this experiment the overall shape of the testing rooms were similar, but opposite in orientation and different in dimension, (room A: width: 10 ft., length: 20 ft.; room B: width: 10 ft., length: 15 ft.) as well as using an identical pool. Based on Clark et al. (2015), which used the exact same rooms and pools as in this experiment, although with different extra-maze cues, it is very possible that head direction information from room A influenced trial 1 of mass training in room B. Despite this initial effect, it was not maintained throughout training, and rats treated with CPP failed to learn the new platform location. This also provides further evidence that NMDAR blockade does not interfere with previously acquired memories.

### Experiment 3

IEG activity is a molecular product of learning and memory related behaviors. Spatial memory tasks such as the water maze have been shown to induce expression of Arc, an IEG, in the hippocampus (Guzowski et al., 2001). IEGs such as Arc are thought to mediate the molecular consolidation of memory because of their time course of action and the types of subsequent molecular mechanisms they induce. When applied to hippocampal tissue, Arc antisense will allow for the induction of LTP, yet its long-term maintenance will disappear (Guzowski et al., 2001). Arc knockout mice similarly express heightened early phase LTP and reduced late phase LTP (Plath et al., 2006). In a fear conditioning paradigm, Czerniawski et al. (2011) showed that an NMDAR antagonist could prevent arc expression in both the dorsal and ventral hippocampus and that arc antisense would impair memory consolidation but spare initial learning, linking NMDARs, Arc, and memory consolidation. Taken together, these electrophysiological, molecular, and behavioral data, all suggest a role for IEGs, and NMDAR dependent Arc in particular, in the long-term consolidation of memory.

For the final experiment, we were interested in assessing immediate early gene (IEG) activity in the hippocampus immediately following mass training in the same versus different rooms. The prediction was that IEGs in hippocampus will be selectively activated in the new room but not the same room condition. We also included a group of rats given mass training in the new room condition but with NMDAR blockade. The prediction is this group would not show the elevations of IEG activity, thus linking NMDAR function and rapid new spatial learning in a new environment.

#### Methods

The design of Experiment 3 is depicted in Figure 1. Behavioral training and testing was very similar to that of Experiments 1 and 2. Pre-training was done in context A for all groups except cage controls. There were four groups in this experiment, differing only in how their mass training was done in reference to pre-training: Same room, New room, New room + CPP, and cage controls (*n* = 5). For the Same room group (*n* = 5), mass reversal training consisted of 16 trials and was performed identically to Experiment 1. For the New room group (*n* = 5) and New room + CPP group (*n* = 5) mass training consisted of 16 trials and was identical to Experiment 2. Drug infusion for the New room + CPP rats was done 30 min before mass training and was performed identical to the CPP group in Experiment 2. The rats were run one at a time during mass training to avoid variation in timing of procedures. After completing mass training, rats were brought back to their home cage until perfusion. Perfusion occurred approximately 70–80 min after trial 8 of mass training. This is a time period in which IEG protein expression after learning is active (Lonergan et al., 2010). Euthanization and perfusion procedures were identical to Experiments 1 and 2.

##### Tissue collection and immunohistochemistry

After extraction, the brains were placed in 4% PFA solution for 24 h, at which point they were then placed in a 30% sucrose + sodium azide solution for a 3-day period of cryoprotection. The brains were then sliced on a freezing microtome in 12 series, to include the entire hippocampal formation. Tissue was stained using a 3-day immunohistochemical protocol that fluorescently stained Arc protein.

On day 1, tissue was pre-washed in 1% PBS solution, 3 × 10 min. Tissue was then placed in 2 ml of a 0.3% triton and PBS solution with a 1:1000 ratio of primary antibody. For Arc protein staining, Arc (c-7) sc-17839 mouse monoclonal IgG (Santa Cruz Biotechnologies) was used. The tissue well was placed on a rotating belly dancer and left to incubate for 24 h. On day 2, the tissue went through a second wash in 1% PBS solution, 3 × 7 min. After the wash, the tissue was placed in a 1:500 solution of PBS and Alexa fluor 488 donkey anti-mouse IgG (H + L) (Thermo Fisher Scientific). The tissue well was then covered in tin foil to prevent light from affecting the staining process. The well was placed back on the belly dancer and the room light turned off and left for a second 24-h incubation period. On day 3, the tissue was put through another 1% PBS wash, 3 × 7 min and then wet mounted onto 1% gelatin and 0.2% chromatin coated slides. The slides were covered and placed in a refrigerator at 3°C for 24 h.

##### Microscopy and cell counts

Cell counts were done on a Zeiss AxioImager M1 microscope (Zeiss, Jena, Germany) using the program Stereo Investigator^®^(MicroBrightField Inc., 2013, Version 10). A constant light intensity exposure was set at 50%. Light was projected through a FITC filter. A 20× magnification was used when counting cells. All labeled granule cells were individually counted in the granule cell layer of the dentate gyrus. All labeled pyramidal cells were individually counted in the pyramidal cell layers of areas CA1 and CA3. Cell counts in each hippocampal slice for each animal were summed and multiplied by 12 (12 series sections were sliced) to get an approximation of total cell number in each region. Tissue photographs were taken and colored with ImageJ^®^(Rasband, 2012). For cell count data, *post hoc* pairwise comparisons were done between groups with Bonferroni correction.

#### Behavioral Results

Over the 4-day pre-training period, the latency for each group followed identical patterns and indicated that all rats from all three groups learned the platform position in the pool. Consistent with this claim, a two-way repeated measures ANOVA showed that there was a significant effect of Trial on latency (*F*_7,84_ = 47.373, *P* < 0.001), no effect of Group (*F*_2,12_ = 3.636, *P* > 0.05) but there was an interaction (*F*_7,14_ = 2.312, *P* < 0.05).

**FIGURE 7:**
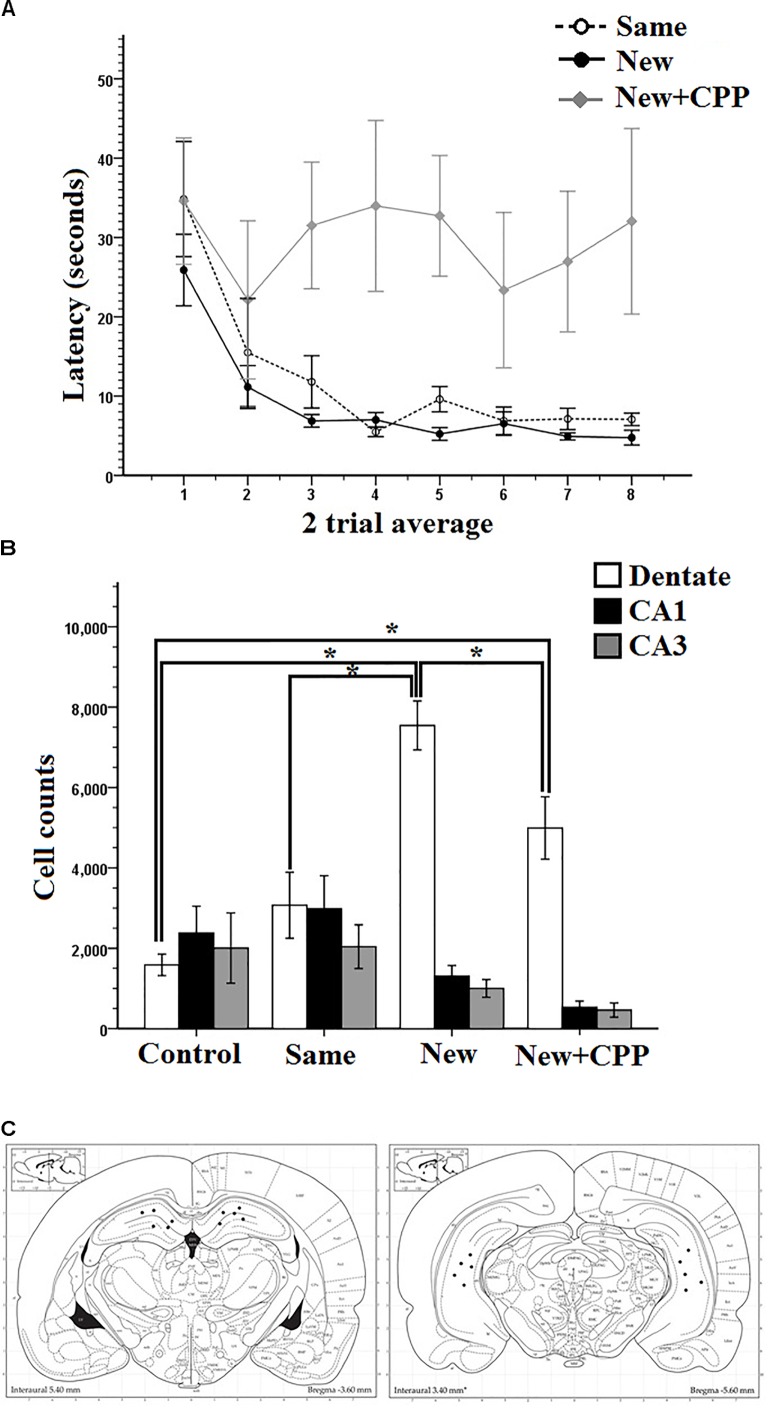
**(A)** Mass training for Experiment 3 after being pre-trained in room A. Same rats were mass trained in room A, New and New + CPP rats were mass trained in room B. Two trial averages of the latency in seconds to find the new platform over training 2 h/16 trials. Same and New rats learned whereas New + CPP rats failed to learn over the course of training. **(B)** Hippocampal cell counts of Arc positive cells in the dentate gyrus, CA1, and CA3, 70–80 min after trial 8 of massed training in Experiment 3. New rats displayed the significantly more Arc in the dentate gyrus than any other group. CPP significantly reduced the expression of Arc in the dentate gyrus in rats mass trained in the room B. **(C)** Dorsal and ventral hippocampal cannulation locations for the CPP rats of group 2 in Experiment 2. Error bars 1 ± SE. ^∗^*p* < 0.05.

Mass-training was analyzed in two trial average blocks (Figure 7A). As can be seen, the average latency of the first trial block was high, at 34.8 s for the Same room rats, 25.9 s for the New room rats, and 34.6 s for the New + CPP rats. By the end of mass training the average latency for the last trial block reduced to 7.0 s for Same room rats, 4.7 s for New room rats, but stayed high at 32.0 s for New + CPP rats. Two-way repeated measures ANOVA revealed a significant effect of Trial on latency (*F*_7,84_ = 4.861, *P* < 0.001) as well as Group (*F*_2,12_ = 7.967, *P* < 0.05) and no interaction (*F*_7,14_ = 1.139, *P* > 0.05). Collectively, these results indicate that both Same room and New room rats successfully learned the platform locations over the course of mass-training while the New + CPP rats did not.

#### Immunohistochemistry Results

Cell counting was done for all rats in areas CA1, CA3, and dentate gyrus. Figure 7B shows that new room rats expressed more Arc positive cells than cage controls, same room rats and New + CPP rats. All three areas were compared between all four behavior groups (Figure 7B). Repeated measures ANOVAs were completed on the data and there were significant effects of area (*F*_2,32_ = 29.428, *P* < 0.001), of Group (*F*_1,16_ = 3.435, *P* < 0.001) as well as an interaction (*F*_6,32_ = 10.712, *P* < 0.001). *Post hoc* pairwise comparisons were made between all the groups and areas. Significant differences were found between cell counts in the dentate gyrus of the New room rats. New room rats expressed more Arc positive cells than cage controls (*P* < 0.001), same room rats (*P* < 0.001), and New + CPP rats as well (*P* < 0.05). The New + CPP rats also expressed more Arc positive granule cells than cage controls (*P* < 0.05) but did not differ from Same room rats. For area CA1 and CA3, no significant differences were found between any of the groups. This indicates that New room training induces more dentate Arc expression than Same room and cage controls, and that intra-hippocampal NMDAR blockade diminishes this effect.

**FIGURE 8:**
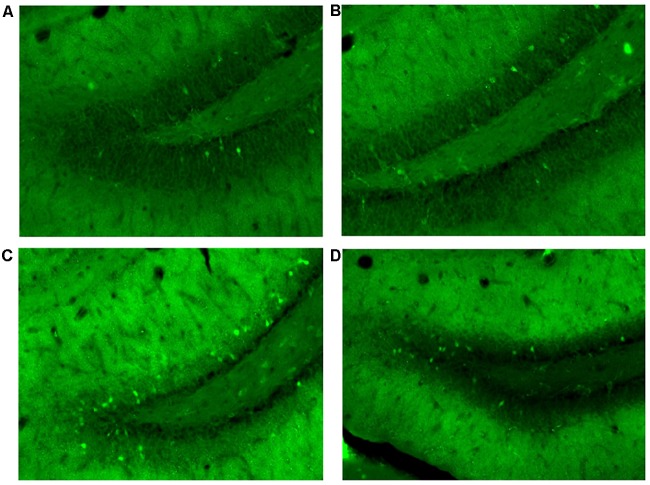
Granule cells in the dorsal dentate gyrus stained positive for Arc protein 70–80 min after mass training in Experiment 3. **(A)** Cage controls. **(B)** Same room. **(C)** New room. **(D)** New + CPP.

Cannulation placement for this experiment can be seen in Figure 7C. Images of the tissue can be seen in Figure 8.

#### Discussion

In this experiment, rats were trained in a version of the MWT that involved multiple training rooms. The effect of the NMDAR antagonist CPP on Arc protein expression in hippocampal sub-regions in response to learning experiences was examined. When pre-trained in the MWT in room A, rats could easily learn a new platform position in room A or in room B. However, when CPP was infused into hippocampus prior to the second training phase, rats could not learn a platform position in room B, despite pre-training. These results are identical to those found in Experiment 2. They further support the idea that hippocampal NMDARs are critical for the acquisition of novel environmental information used in spatial navigation.

The expression of the protein for the immediate early gene Arc differed between group and region, depending on the training conditions. In the dentate gyrus, expression was high for the New group compared to the equally low cage controls and Same room group. When CPP was given, this corresponded with both a reduction in dentate Arc expression and an impairment in learning compared to the New room group. No significant differences were observed for either area CA1 or CA3 between any of the groups.

Despite a lack of a defined pattern of behavior related activity in areas CA1 and CA3, the pattern of Arc expression in the dentate gyrus fits with the behaviors exhibited by the rats in their respective learning conditions. Learning a new spatial platform location in the same environment as pre-training, dentate arc expression was not significantly different from cage controls. Learning in the new room, however, produced a large increase in dentate Arc expression. This would indicate that if Arc is necessarily tied to learning and plasticity, that the same room reversal training in the MWT is not the same as novel room training, behaviorally or molecularly.

## General Discussion

In these experiments, we assessed the role that hippocampal NMDARs have in spatial memory and the molecular processes associated with it. Without the use of NMDAR function in the hippocampus, rats were capable of expressing what they had learned previously, and they could also learn new spatial information in the same environment as pre-training. When brought to a completely novel training environment, rats could not learn new spatial information at all without the use of hippocampal NMDARs. These results lead to the conclusion that NMDARs mediate the acquisition of novel environmental information to be used in spatial navigation.

This type of NMDAR dependent behavior was also found to induce specific patterns of Arc expression in the hippocampus, namely the dentate gyrus. Expression was low in the NMDAR independent task (same room), and high in the NMDAR dependent task (new room). CPP both blocked learning and reduced Arc expression in animals trained in the new room. This pattern of results points to a link between these three phenomena: NMDAR function, Arc expression, and novel spatial learning. The relationship between these will be explored below.

### NMDAR Blockade in the Dorsal Versus Full Hippocampus

The inspiration and design of the current experiments came from McDonald et al. (2005). In that series of experiments only the dorsal hippocampus was blocked for the intracranial experiments and no effect of NMDAR blockade on mass-training of a new spatial position was reported. Experiment 1, from the current work, was completed to determine if the pattern of results observed in the McDonald et al. (2005) study was due to functional NMDARs in the ventral hippocampus. The ventral portion of the hippocampus has been shown to support certain aspects of spatial learning (Ferbinteanu et al., 2003). Very few MWT studies have been done with full hippocampal NMDAR blockade (Steele and Morris, 1999; Inglis et al., 2013). Both the experiments cited above utilized pre-training procedures followed by further training under receptor blockade, and both experiments resulted in successful spatial learning under specific circumstances, despite full blockade. These experiments will be explained in more detail below.

As a stated hypothesis in Experiment 1, the full hippocampal NMDAR blockade discounts the possibility that the reason why animals were capable of learning novel spatial locations in the McDonald et al. (2005) study was due to a functional ventral hippocampus.

One important difference between the results reported in Experiment 1 and those from the McDonald et al. (2005) study was the 8- and 24-h probe results. In the original study, control rats show a clear quadrant preference for the new platform location 24 h later while CPP rats did not. In the present study, the strength of the representation following these delays seemed to have been compromised somewhat. This difference from the McDonald et al. (2005) paper might be because we instituted, in the present set of experiments, the 30-s probe trial immediately after mass training. This did not happen in the original 2005 study. This probe can lead to some extinction and provides the subjects with the knowledge that the platform might be located nowhere (probe trial) or elsewhere (mass training). It is possible that the controls transition from new location, old location, and other quadrants more quickly which could negatively affect the quadrant preference measure. Further research is required to confirm this hypothesis.

### NMDARs and Memory Retrieval

The lack of NMDAR involvement in memory retrieval has long been accepted after several experiments outlining rats’ ability to use previously acquired information during NMDAR blockade (Shapiro and Caramanos, 1990; Bast et al., 2005). Biologically this would make sense, if NMDARs are necessary for LTP induction and learning based plasticity, then once the circuit connections representing the memory have been formed and consolidated, plasticity mechanisms should not be involved in simple circuit activation during retrieval. However, recent evidence has shown the contrary; that NMDAR blockade can prevent the expression of previously established fear memories (Lopez et al., 2015). Our results in Experiment 1 further support the claim that NMDARs are not involved in memory retrieval and expression. When analyzed as a probe, trial 1 of mass training revealed that rats from both groups displayed a spatial preference for the pre-training platform location, indicating that it was both successfully learned and retrieved.

### NMDARs and Spatial Learning

Starting with Bannerman et al. (1995) and Saucier and Cain (1995), the idea of NMDAR independent learning began to be explored. Novel MWT training in the presence of NMDAR antagonist drugs resulted in learning impairments, but by changing certain parameters of the task, these impairments could be eliminated. Since then, many studies have examined this phenomenon, through NMDAR independent extinction (Santini et al., 2001), place cell formation (Kentros et al., 1998), LTP (Grover and Teyler, 1990), and molecular cascades associated with learning and plasticity (Moosmang et al., 2005). How exactly learning processes can be made NMDAR independent is not certain. Some have shown that behavioral strategies are learned once, and then can be applied across multiple learning scenarios independent of NMDAR plasticity (Hoh et al., 1999). Others have argued that where in the brain learning takes place changes over training (Inglis et al., 2013).

Almost all studies examining NMDAR independent spatial learning have done so using pre-training, in some form or another. A review of the literature on experiments using NMDAR blockade, pre-training, and spatial learning reveals two overall findings. The first is spatial learning can be made NMDAR independent when done with the same information available to prior learning and is also done rapidly. This is made most clear by Steele and Morris (1999) where rats were pre-trained in the MWT on a version that involved a new platform location every day. After several days, rats continued training in the same room but with hippocampal NMDAR blockade. The manipulated variable was inter-trial interval, and it was found that when it was kept short at 15 s rats learned successfully. However, when it was lengthened to 20 min, they failed to learn. This means that rapid learning was possible while learning over longer periods of time was not. Because the platform changes every day, the effect of receptor blockade on long-term training cannot be assessed.

Work done by Saucier and Cain (1995) and Hoh et al. (1999) have shown that non-spatial pre-training (NSP) can lead to rapid NMDAR independent spatial learning. The argument put forth by these researchers is that NSP allows for behavioral search strategies to be developed prior to spatial training and that this is what leads to NMDAR independence later on. However, behavioral search strategies themselves were also found to be learned independent of NMDAR. This contrasts with earlier work done (Morris et al., 1990) in which NSP led to impairments in same room spatial training that was done over the course of several days. NSP is performed with a curtain around the pool and so may potentially conflict with our explanation of previously learned environmental information hypothesis. However, the complete environmental repertoire that contributes to spatial navigation behaviors goes beyond wall cues like posters.

The MWT environment can be divided into two separate spaces: the intra-maze (local) environment which contains everything inside the pool walls, and the extra-maze (global) environment which contains everything outside the pool walls but inside the experiment room. Some evidence shows that aspects of spatial navigation can even transfer between multiple contexts and so would constitute potentially larger, environment independent navigation strategies as well, such as heading direction (Taube and Burton, 1995; Clark et al., 2015). Given the continued use of the maze and global location of the maze throughout training, NSP does not necessarily mean that no usable environmental information is used to learn the platform location. With this in mind, in the studies using NSP, rats successfully learn the MWT when trained rapidly (Saucier and Cain, 1995; Hoh et al., 1999).

Our work reinforces these conclusions. It shows that in the absence of NMDAR function in the hippocampus, rapid learning is possible when the extra-maze, environmental information used to learn a new escape location is identical to what has been previously learned. After both an 8-h and a 24-h retention period, everything that was learned during mass training was forgotten. Our specific pattern of results indicates that whatever was learned was not consolidated. A lack of consolidation may explain why rats in these types of tasks can learn rapidly but not with increased ITIs or over several days. NMDAR involvement in memory consolidation has been shown on multiple occasions (Santini et al., 2001; McDonald et al., 2005).

The second finding, in the literature, is that novel spatial learning can be made NMDAR independent when done over several days. This comes primarily from two experiments. Bannerman et al. (1995) was able to get rats to learn the MWT in a room different from that of pre-training, and did so over multiple training days. A similar effect was found in that experiments replication (Inglis et al., 2013). In the Bannerman et al. (1995) study, a NSP experiment was also done, and when done in room 1, it did not allow for spatial learning in room 2 across multiple days. NSP is a very specific type of pre-training that eliminates extra-maze cues. Given that there is no visual environmental information outside of the pool, it is possible that the rats given NSP pre-training treated room 2 in the Bannerman study in the same way that the rats in the Cain and Hoh studies treated their respective training rooms. Stated differently, without previous environmental information to compare to new information, all information is new, and so new training done in a different or same room will not matter. A similar intra-maze environment and lack of extra-maze specific cues means that there is no such thing as same room or different room (dependent on extra-maze cues) following NSP, and that all subsequent spatial training will follow the same principles. This would also explain the multiple day learning impairments observed in Morris et al. (1990).

However, multiple day NMDAR independent learning does not apply to naïve rats in the MWT. This may be because novel MWT training does not just involve spatial learning but also developing successful motor patterns for swimming, trying out search strategies, as well as reducing fear. The MWT is an aversive learning task and given NMDAR involvement in motor function and anxiety, some have argued that learning deficits cannot be dissociated from other types of effects (Keith and Rudy, 1990; Ahlander et al., 1999). Because of the ubiquitous nature of the NMDAR, it may have a role in many neural and behavioral processes outside of spatial navigation and learning. This is why pre-training is used, it allows for specific aspects of the task to be isolated and examined.

In all of the work involving MWT training that relies on previously learned environmental information, NMDAR independent learning is successful when done rapidly but not when done with increased ITIs or over several days. However, when training is done with novel environmental information and the potentially confounding influence of behavioral strategies, anxiety, and sensori-motor impairments are removed, learning may be NMDAR independent when done over several days, but not rapidly. To our knowledge, no one has examined the role of NMDAR in rapid novel spatial learning after pre-training. Our work provides support for these conclusions by showing that when transferred to a new room with novel environmental cues rats without NMDAR function cannot rapidly learn.

The shift in NMDAR dependence may have to do with the requirements of the task. Reversal learning in the MWT consists of fundamentally different task parameters and learning requirements than novel learning. When put through the MWT for the first time, rats must learn many things ranging from behavioral search strategies, spatial locations, and interacting with the maze, the experimenter, and the environment. These include, but are not limited to, swimming away from the pool wall, the size and stability of the hidden platform, the arrangement of extra-maze cues, etc. One of the main reasons why pre-training procedures are used is to limit the type of learning that must occur in subsequent training procedures for successful maze navigation. When put through reversal learning in the same spatial context after a pre-training procedure, the only thing required for the rat to learn is a new platform position. The dimensions and details of the maze and room, as well as a successful search strategy, have already been learned. Also, rats have to extinguish the previously learned spatial information, so that reversal doesn’t just involve new learning, but extinction as well. The rat must do this using information and strategies it is already familiar with.

Further training with a completely new set of information such as in Experiment 2 is also different from both naïve training and same room reversal. Several components of training are already familiar to the rat such as swimming and an expectation of the rules of further training. Yet some things are completely unfamiliar like where the animal is and what exists in its environment. In this way, three different stages of MWT training, naïve, new room, same room reversal, all have increasing amounts of familiarity to them, respectively.

This means that all three stages of training may involve different search strategies, or the same strategies but based on different types of available information. Given the different task requirements during each stage, it is not unlikely that the role plasticity has will vary across stages of training. Our own results, as well as a review of the relevant literature, reveal that NMDARs in the hippocampus will be heavily involved in several neural and behavioral processes outside of learning during naïve MWT training, resulting in an inability to dissociate between learning, anxiety, and sensori-motor processes just from behavioral data alone. After extensive pre-training and task-familiarization, NMDARs consolidate changes to existing spatial information. And that when processing completely novel environmental information, are necessary for its rapid learning.

### Immediate Early Genes and the Dentate Gyrus

The use of IEGs in neuroscience research is commonly used to map behaviorally relevant neural activity. The IEG Arc is thought to be dependent on LTP related activity and so is used to map neurobiological mechanisms of learning and memory. Learning and memory behaviors are known to reliably induce Arc mRNA and protein expression in behaviorally relevant brain regions. Activation of the NMDAR has been identified as a critical mechanism that leads to Arc expression. Blocking NMDARs with antagonist drugs reduces behavior induced Arc mRNA (Czerniawski et al., 2011). Furthermore, NMDAR hypofunction leads to reduced Arc expression (Balu and Coyle, 2014). The links between learning, NMDAR, LTP, and Arc activity is well supported by research (Guzowski et al., 2001; Shepherd and Bear, 2011).

The results from Experiment 3 show that in one version of a MWT that did not require NMDARs to learn, Arc protein expression was not significantly different from cage controls. In a version of the task that did require NMDARs to learn, Arc protein expression was high compared to cage controls and the NMDAR independent task. When an NMDAR antagonist was introduced to the hippocampus, it impaired learning as well as reduced Arc protein expression. The level of expression in the CPP group was significantly below the New room group but also significantly higher than the cage control group. No observable patterns emerged in areas CA1 or CA3, however, the pattern of expression described above was observed in the dentate gyrus.

Two important conclusions can be reached from these results. One is that NMDAR dependent learning of novel environmental information activates Arc in the dentate gyrus. The other is that NMDAR blockade reduces Arc expression in the dentate gyrus. Because we did not determine the binding efficacy of CPP administered, it is impossible to know if the heightened Arc expression seen in the CPP + New group relative to cage controls was because of NMDAR independent processes or due to some residual NMDAR function. We believe the latter is the most likely explanation, which likely indicates that the intra-hippocampal CPP injections resulted in extensive but not complete NMDAR block. Further research is required to assess this and other potential explanations.

Given the specific pattern of results our experiments produced, as well as the contributions of other work on this topic, we can also make hypotheses regarding the function of dentate gyrus plasticity as it pertains to spatial learning.

Dentate specific lesions of granule cells using Colchicine injections (Sutherland and Rudy, 1988) have been shown to impair both reference memory across training days and working memory within training days in spatial water tasks (McNaughton et al., 1989; Xavier et al., 1999). Selective genetic knockout of NMDAR NR1 subunit exclusively in the dentate gyrus of mice produces working memory impairments while maintaining reference memory in an 8-arm radial maze (Niewoehner et al., 2007). Similarly, McHugh et al. (2007) produced a dentate specific knockout mouse that could learn context fear conditioning. However, the mice could not learn context discrimination, supporting the dentate’s proposed role in pattern separation (Leutgeb et al., 2007; Kesner, 2013). Chen et al. (2009) produced a mutant mouse with reduced GluN1R subunit expression in the dentate gyrus. The behavioral phenotype of these mice was that of impaired MWT performance over multiple days, as well as impaired same room reversal learning over multiple days. The results from lesion and knockout experiments points to three functions of the dentate gyrus: (1) its role in the hippocampal circuits mediating spatial learning; (2) a role in both working memory with a sparing of reference memory; and (3) supporting discriminations between multiple learned environments. Interestingly, genetic experiments show a very specific type of behavioral learning deficit after NMDAR manipulation in the dentate gyrus, namely, the inability to discriminate environmental information and learn rapidly where as in our experiment, rapid novel environmental learning resulted in NMDAR dependent activity in the dentate gyrus. The previous work produces behavioral effects after the molecular manipulation, our work shows a possible molecular effect after similar behavioral manipulations. Although, more work would necessarily have to be done to prove it, this bidirectional effect demonstrates a link between NMDAR plasticity in the dentate gyrus and the rapid learning of novel environmental information.

### Potential Limitations

One potential caveat when interpreting the pattern of effects from the current experiments is trying to differentiate between memory consolidation versus extinction effects of the NMDAR manipulation. Rats given CPP during mass-training in the same room showed a spatial preference for the pre-trained platform location during the 24-h probe. It could be argued that this happens because NMDAR blockade prevented extinction learning from occurring toward the original platform. When learning a new platform location after pre-training, presumably two types of learning are going on: new spatial learning and extinction learning for the old platform position. However, it is very unlikely that rats would be able to learn a new spatial location during training while also not being able to learn extinction for an old spatial location at the same time since the mechanisms underlying cellular consolidation of extinction memories are proposed to be very similar to the cellular consolidation of other memories (Santini et al., 2001; Quirk and Mueller, 2008).

Another potential limitation with our claim of a role of NMDAR receptors in hippocampal memory consolidation is that the results of the 8- and 24-h probes in Experiment 1 may be due to state dependent learning during mass training. The fact that CPP rats in Experiment 1 behaved differently 24 h after mass training compared to immediately after could be due to the fact that the immediate probe happened while CPP was still active in these rats, and not 24 h later. However, prior work investigating NMDAR involvement in consolidation suggests that it is not state dependent (Kentros et al., 1998; Santini et al., 2001; McDonald et al., 2005).

Many studies examining the effects of spatial learning on IEG expression have found it in all three sub-regions of the hippocampus proper: Dentate gyrus, CA1, and CA3 (Guzowski et al., 1999, 2001). However, in our experiment, same room reversal training did not induce Arc expression any different from cage controls. New room training did induce higher Arc expression but was limited to the dentate gyrus. An alternative explanation of this effect could be the time course of protein action. It is possible, although has not been explored to our knowledge, that IEG protein expression varies throughout the hippocampus after MWT training in a time dependent manner. For example, using our paradigm, if animals were euthanized 70 min after mass training trial 1 versus after trial 16, then sub-region expression differences may be seen. Consistent with this idea, it has been shown that rats change their search strategies throughout the course of training (Graziano et al., 2003). Different strategies may induce activity in different hippocampal sub-regions depending on what the animal is learning. However, given the robust time course of Arc protein action (30–90 min), sub-region activation dependent on training time would likely be observed regardless of which trial the rat was perfused in our experimental design.

### Contributions of the Present Work

The novel contributions of this work center on three main findings. The first is that hippocampal blockade across both dorsal/ventral aspects results in similar behavioral patterns in the MWT as a dorsal-limited blockade. This suggests that the lack of an impairment of NMDAR dorsal hippocampal blockade on rapid acquisition of a new spatial location, reported in McDonald et al. (2005) was not due to intact ventral hippocampal NMDA function. Second, NMDARs appear to be necessary for encoding and consolidation of new environmental information that will be utilized for spatial navigational behaviors but not new spatial navigational behaviors in a previously trained context. Finally, NMDAR dependent Arc activation occurs in the dentate gyrus after rapid spatial learning in a new environment. These three novel contributions to the understanding of the neurobiology of learning and memory will hopefully lead to more discoveries in the future.

## Author Contributions

RJM was the principal investigator, provided study design and funded the research with his grants, and wrote the article. CB provided study design, collected the data, performed the statistical analysis, rodent surgery, microscopy, and wrote the article.

## Conflict of Interest Statement

The authors declare that the research was conducted in the absence of any commercial or financial relationships that could be construed as a potential conflict of interest.

## Supplementary Material

FIGURE S1 Pretraining for groups 1 and 2 in Experiment 1. Four trial averages of the Path length in meters to find the platform over training 4 days/eight trials per day. Both controls and pre-CPP rats successfully learn over training. Also shows mass training for groups 1 and 2 in Experiment 1. Two trial averages of the Path lengths in meters to find the new platform over training 2 h/16 trials. Both controls and CPP rats learn over training.

FIGURE S2 Pretraining for groups 1 and 2 in Experiment 2. Four trial averages of the Path length in meters to find the platform over training 4 days/eight trials per day. Both controls and pre-CPP rats successfully learn over training. Also shows mass training for groups 1 and 2 in Experiment 2. Two trial averages of the Path lengths in meters to find the new platform over training 2 h/20 trials. CPP rats fail to learn over mass training.
