# Supplementary figures and images for: A Specific Role of Hippocampal NMDA Receptors and Arc Protein in Rapid Encoding of Novel Environmental Representations and a More General Long-Term Consolidation Function
Source: Front Behav Neurosci. 2019 Feb 26;13:8. doi: 10.3389/fnbeh.2019.00008 (PMC6399163; doi:10.3389/fnbeh.2019.00008)

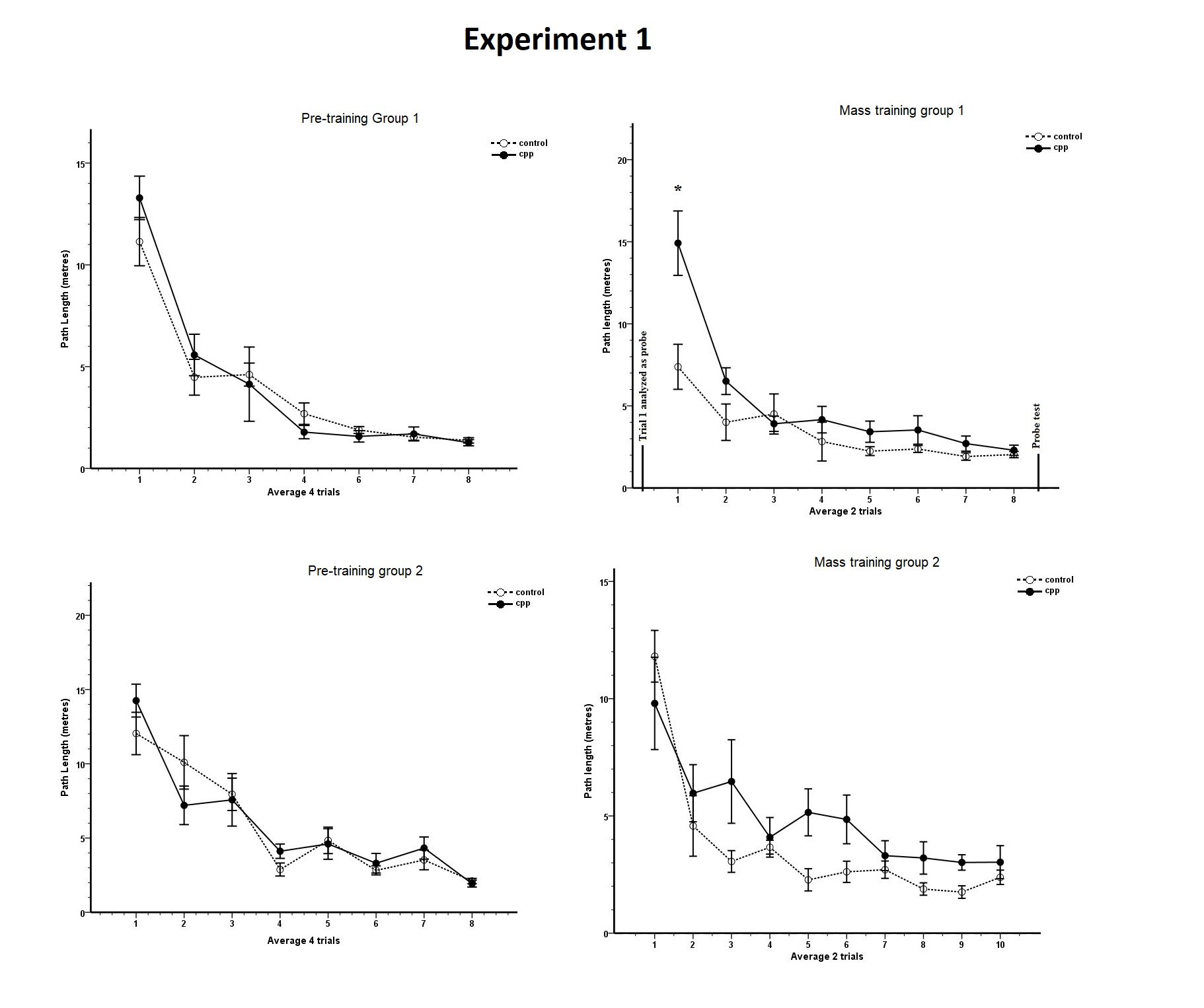

Supplement: FIGURE S1 — Pretraining for groups 1 and 2 in Experiment 1. Four trial averages of the Path length in meters to find the platform over training 4 days/eight trials per day. Both controls and pre-CPP rats successfully learn over training. Also shows mass training for groups 1 and 2 in Experiment 1. Two trial averages of the Path lengths in meters to find the new platform over training 2 h/16 trials. Both controls and CPP rats learn over training. [file Image_1.TIF]

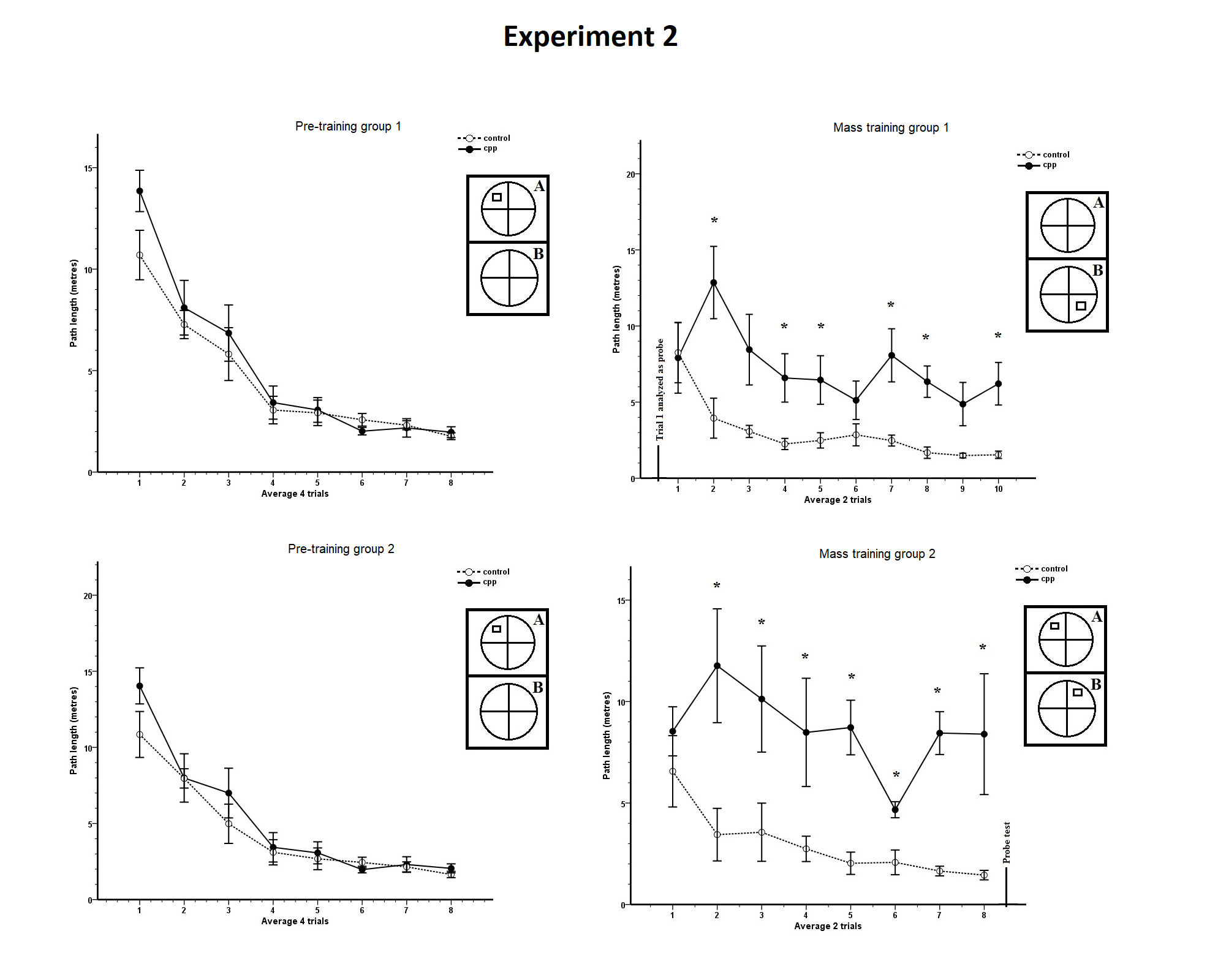

Supplement: FIGURE S2 — Pretraining for groups 1 and 2 in Experiment 2. Four trial averages of the Path length in meters to find the platform over training 4 days/eight trials per day. Both controls and pre-CPP rats successfully learn over training. Also shows mass training for groups 1 and 2 in Experiment 2. Two trial averages of the Path lengths in meters to find the new platform over training 2 h/20 trials. CPP rats fail to learn over mass training. [file Image_2.TIF]
